# Editorial: Frontiers in Language Assessment and Testing

**DOI:** 10.3389/fpsyg.2021.691614

**Published:** 2021-05-28

**Authors:** Vahid Aryadoust, Thomas Eckes, Yo In'nami

**Affiliations:** ^1^National Institute of Education, Nanyang Technological University, Singapore, Singapore; ^2^TestDaF Institute, Ruhr University Bochum, Bochum, Germany; ^3^Faculty of Science and Engineering, Chuo University, Hachioji, Japan

**Keywords:** comprehension, structural equating modeling, vocabulary, working memory, quantitative method, Sign Language, interpreting, scientometric analysis

Although language assessment and testing can be viewed as having a much longer history (Spolsky, 2017; Farhady, 2018), its genesis as a research field is often attributed to Carroll's (1961) and Lado's (1961) publications. Over the past decades, the field has gradually grown in scope and sophistication as researchers have adopted various interdisciplinary approaches to problematize and address old and new issues in language assessment as well as learning. The assessment and validation of reading, listening, speaking, and writing, as well as language elements such as vocabulary and grammar have formed the basis of extensive studies (e.g., Chapelle, 2008). Emergent research areas in the field include the assessment of sign languages (Kotowicz et al., 2021). In addition, researchers have employed a variety of psychometric and statistical methods to investigate research questions and hypotheses (see chapters in Aryadoust and Raquel, 2019, 2020). The present special issue entitled “Frontiers in Language Assessment and Testing” set out to shed light on these advances and approaches in the field of language assessment.

We received a number of proposals, 13 of which were ultimately accepted for publication in the special issue. Five major themes emerge from the accepted papers: (i) the quantitative perspectives of the history and evolution of language assessment as presented in the scientometric study by Aryadoust et al., (ii) the issues surrounding the assessment of listening and reading comprehension discussed in the five papers by Spoden et al.; Cai; Wallace and Lee; He and Jiang and Hamada., (iii) the assessment of speaking and writing proficiency in the two papers by Fan and Yan and Li et al., (iv) the assessment of sign languages and interpreting competence in the three papers by Rosenburg et al.; Hall; and Wang et al., and (v) the use of advanced quantitative methods presented in the two papers by Koizumi and In'nami and Dunn and McCray.

## Quantitative Perspectives of the History and Evolution of Language Assessment: A Scientometric Study

Aryadoust et al. presented an extensive scientometric review of 1,561 articles published in the “core” language assessment journals and 3,175 articles published in the general journals of applied linguistics. Using a document co-citation analysis (DCA) technique, they found that publication in the core journals primarily focused on the assessment of the four language skills (listening, speaking, reading, and writing), while there were fewer papers that examined washback, feedback, and corpus linguistics topics. Similarly, the assessment research in the general journals also focused on the assessment of oral proficiency, vocabulary, writing, reading, and grammar, while fewer publications investigated topics related to cognition and knowledge. These topics included memory, affective schemata, awareness, semantic complexity, and explicit vs. implicit language knowledge. Interestingly, no assessment instruments with entire validity arguments formed the basis for the majority of the studies. This was consistent with findings from previous studies whose authors argued that “collecting such evidence to establish an all-encompassing validity argument is an arduous and logistically complex task” (p. 3). Aryadoust et al. suggested that minimum requirements for examining the validity of tests would include reliability and psychometric evidence to show that the tasks or items functioned properly while evaluating the construct that the test set out to measure.

## Assessment of Listening and Reading Comprehension

Spoden et al. investigated the effect of in- and out-of-school language learning opportunities and exposure to media on the correlation between listening and reading skills over time (i.e., the start and end of secondary schooling) in a bilingual pre-tertiary population in Germany. Pre-tertiary populations, as the authors rightly argue, have not drawn the attention of researchers in language assessment as much as adult second language learner populations have. Thus, the study addresses a wide gap in knowledge. Using the latent regression Rasch models and correlation analysis, Spoden et al. found evidence for a converging pattern of growth common between listening and reading. They further reported that this finding was consistent across language learning groups with different backgrounds such as learners with varying experiences in extracurricular English-learning programs. Some theoretical studies have postulated that the auditory modality of the language input in listening could disadvantage L2 learners in listening comprehension compared with visual input in reading, as auditory input is transitory (Aryadoust, 2019). In light of this, Spoden et al.'s study indicates that “modality specificity becomes a less important factor to affect comprehension test scores at the end of secondary education in Germany” (p. 4). The authors called for further research to consider how vocabulary and grammar, for example, affect listening and reading, a topic that Cai partially addressed.

Cai investigated the relationship between lexical and semantic knowledge with listening proficiency in academic tests of listening comprehension, using “auditory receptive tasks contextualized in natural discourse” (p. 1). The study used several tasks to operationalize and measure the relationship between listening and language elements, comprising partial dictation, an auditory receptive task, and a standardized listening test, that were administered to a sample of 258 college-level English learners in China. Hierarchical regression analyses revealed that the lexical and semantic knowledge of the participants explained a large proportion of variance (62%) in the listening test scores. The author calls for further studies of the relationship between listening and the language elements investigated to improve the generalizability of the results across different contexts.

In another study of the assessment of listening comprehension, Wallace and Lee investigated the effect of vocabulary size alongside executive functions (EFs) on L2 listening comprehension. The study began from the assumption that language components such as vocabulary and grammar have a significant effect on listening comprehension, yet as language proficiency increases, other factors such as EFs of working memory start to play a crucial role in comprehension. In this study, EFs were operationalized as shifting (“switching attentional focus among mental representations”) and updating (“revising information held in temporary storage”) (p. 1). Using structural equation modeling (SEM), the authors found no main effects or moderation effects of EF, while vocabulary size remained a significant predictor of listening. These results show that, as the authors hypothesized, vocabulary knowledge remains the most important predictor of listening ability, whereas non-linguistic factors such as EF do not contribute to the listening ability of less capable L2 learners.

He and Jiang conducted an extensive review of L2 listening research in 87 studies in peer-reviewed journals and research report series published between 2001 and 2020. The authors used a socio-cognitive validity framework, which consisted of cognitive validity, criterion-related validity scoring validity, context validity, test-taker characteristics, and consequential validity (Weir, 2005). By examining the content of the studies based on their coding scheme, the authors identified 13 research themes in relation to the six components of validity in Weir's (2005) framework. For example, the authors reported that 94.25% of the examined studies focused on context validity, cognitive validity, test-taker characteristics, and scoring validity. In their focus on cognitive ability, however, they included eye tracking and brain activation research. The authors also found that task development, task output/input, and speaker characteristics received “considerable attention” in context validation, whereas there was a dearth of research focusing on consequential and criterion-related validity.

Hamada was interested in the effects of extensive reading instruction on reading comprehension. In Study 1, the author collected previous studies, calculated effect sizes, and grouped them according to their study features. Although instruction was effective overall (Cohen's *d* = 0.55 [95% confidence interval = 0.39, 0.70]), it was less so when only examining studies that had control and treatment groups of equal reading proficiency (*d* = 0.37 [0.24, 0.50]). This suggests the importance of ensuring group equivalency before interpreting instruction effects, which otherwise tend to be overestimated. Study 2 examined whether the estimated instruction effect size from the meta-analysis in Study 1 would be reproducible in an actual classroom study. After analyzing data from 109 learners using propensity score methods, the results suggest that the instruction was effective, with an effect size concurring with that estimated in Study 1. These results from Studies 1 and 2 highlight the importance of evidence-based teaching in the classroom.

## Assessment of Speaking and Writing Proficiency

Fan and Yan conducted a narrative review of papers published in two journals in language assessment—*Language Assessment Quarterly* and *Language Testing*. A total of 104 papers on speaking assessment were classified under the six types of inferences in an argument-based validation framework (Chapelle, 2008). Nearly half of the papers (40.38–48.08%) concerned evaluation, generalization, and/or explanation inferences, with a few (3.85–6.73%) addressing domain description, extrapolation, and/or utilization inferences. The most frequently researched topics included (a) speaking constructs, (b) rater effects, and (c) factors that affect test performance. The studies often used quantitative methods (e.g., analysis of variance, Rasch measurement) to examine questions that would pertain to the evaluation and generalization inferences, and qualitative methods (e.g., discourse analysis, interview) to examine questions that would pertain the explanation inference. The authors conclude that more research on domain description is necessary, particularly in relation to language assessment for specific purposes. They also place importance on taking not only a psycholinguistic but also a sociocultural approach to understand the construct of speaking ability more comprehensively.

Although score differences among subgroups have been examined using differential item functioning (DIF) analysis, it is not always easy to interpret such differences substantively. To address this issue, Li et al. focused on score differences between male and female learners in a standardized writing assessment. The writing prompt was found to favor females, although negligibly so. They investigated the source of this difference using 123 linguistic features. Two cohesion features and four syntactic features correlated significantly with writing scores. As the direction of these correlations was mixed (positive or negative) depending on features, their impacts on writing scores could be offset, producing a negligible gender difference in writing test scores. Other studies could also combine DIF analysis with linguistic analysis to gain a better understanding of the factors that affect test performance.

## Assessment of Sign Languages and Interpreting Competence

With the aim of measuring deaf children's literal and inferential understanding of passages, Rosenburg et al. developed an assessment tool called the American Sign Language Text Comprehension Task. They conducted a validation study administering the tool to deaf children of deaf parents and deaf children of hearing parents. Results showed that the internal consistency, discriminability, and difficulty of the instrument were acceptable. Scores correlated significantly with those of synonym and antonym tests. Deaf children of deaf parents scored better than deaf children of hearing parents, a pattern that was consistent with earlier findings. Taken together, these results provide positive evidence for the validity of the new assessment tool and suggest its utility as a measure of text comprehension skills in deaf children.

Language assessment research has typically focused on language outcomes, providing information about examinees' vocabulary knowledge, grammar skills, or speaking proficiency. Much less attention has traditionally been devoted to language input. When targeting language knowledge of deaf and hard-of-hearing (DHH) children, Hall argues that the assessment scope needs to be significantly broadened. His detailed conceptual analysis draws our attention to the manner in which DHH children address language input, which is truly diverse, and calls for developing measures reflecting the language input that DHH children received during infancy and toddlerhood. Hall outlines several features required of such measures. These include examining an aggregated picture of how a DHH child has interacted with language input over a precisely defined period and representing the extent to which a DHH child has had limited access to language input, finally yielding more informative profiles of language access. Such profiles, in turn, can help inform language assessment at both the individual and population levels. At the individual level, suitable language input measures could distinguish between DHH children's language delay and language disorder. At the population level, such measures could be useful in understanding how language relates to child development.

In another study, Wang et al. reported on the development of the Chinese Standards of English-Interpreting Competence Scales. This is a standardized, national framework of Chinese-English interpretation competence that can be used to train and assess interpreters in China. The project consisted of (i) the definition of interpretation competence, (ii) the relationship between the definition and task, (iii) the collection and analysis of descriptors, (iv) quantitative validation, and (v) qualitative validation. Initially, the authors collected or created 9,208 descriptors. Quantitative and qualitative analyses of data from surveys and interviews reduced and refined the initial pool of descriptors to 369 descriptors. The authors argued that descriptors could be used to create tasks and teaching materials for classroom use, as well as self-assessment.

## Use of Advanced Quantitative Methods

Studies of the strength of the relationship between vocabulary size and vocabulary depth have yielded mixed findings. It is therefore not clear whether vocabulary knowledge is a single construct incorporating both size and depth or else two separate constructs of size and depth. To address this issue, Koizumi and In'nami analyzed vocabulary test data from 255 Japanese learners of English. Results of conventional and Bayesian structural equation modeling suggest that vocabulary size and depth are two closely correlated (*r* = 0.946 and 0.943 for conventional and Bayesian analyses, respectively) but separate abilities. This suggests that a comprehensive measurement of vocabulary knowledge requires an assessment of both size and depth. The results can be reported as a composite score of vocabulary knowledge, or two separate scores of size and depth.

Within the framework of structural equation modeling, Dunn and McCray examined the role of the bifactor model, where a general factor and a specific factor explain an observed variable. This is important in language assessment, as the structure of a test relates to how the scores are reported. To demonstrate this, they analyzed data on the grammar and vocabulary sections of the British Council's Aptis test using a bifactor model, a correlated-factor model, and a unidimensional model. The bifactor model explained the data best, suggesting the possible reporting of either a composite score or skill-specific scores. However, the average size of factor loadings was similar across models, suggesting the sufficiency of simply reporting a composite score. The authors conclude by reporting a composite score, a practice consistent with the Aptis test.

Finally, yet importantly, we would like to thank the reviewers for their valuable comments and suggestions. Without their help, the publication of this special issue would not have been possible. We hope that the readers of the journals will find this collection of research papers useful.

## Author Contributions

VA, TE, and YI contributed to conception and writing of the editorial. All authors contributed to the article and approved the submitted version.

## Conflict of Interest

The authors declare that the research was conducted in the absence of any commercial or financial relationships that could be construed as a potential conflict of interest.
